# Complete genome sequences of Cluster FE *Arthrobacter globiformis* phages Piku and Utopia

**DOI:** 10.17912/micropub.biology.001903

**Published:** 2025-12-16

**Authors:** Sandra Labib, Ayat Hafeez, Hajira Choudry, Rida Ali, Kanza Hussain, Esbeida Olascoaga, Mohammad Khan, Yousuf Kamal, Subohi Fatima, Nikola Slakeski, Manal Syeda, Ibrahim Muhammad, Bisma Khan, Tiara Pérez Morales

**Affiliations:** 1 Biological Sciences, Benedictine University, Lisle, Illinois, United States; 2 Biological Sciences, College of DuPage, Glen Ellyn, Illinois, United States; 3 Biological, Physical, and Health Sciences, Roosevelt University, Chicago, Illinois, United States

## Abstract

Phages Piku and Utopia were isolated from soil samples in Illinois, USA, on *A. globiformis* B-2979 and B-2880, respectively. Both phages have genomes encoding 22 genes, including an endolysin. The genomes are highly conserved, differing by only three genes, including genes involved in structural and replication functions, and one of unknown function. Based on gene content, both phages are assigned to cluster FE.

**Figure 1. Plaque and viral morphologies:**
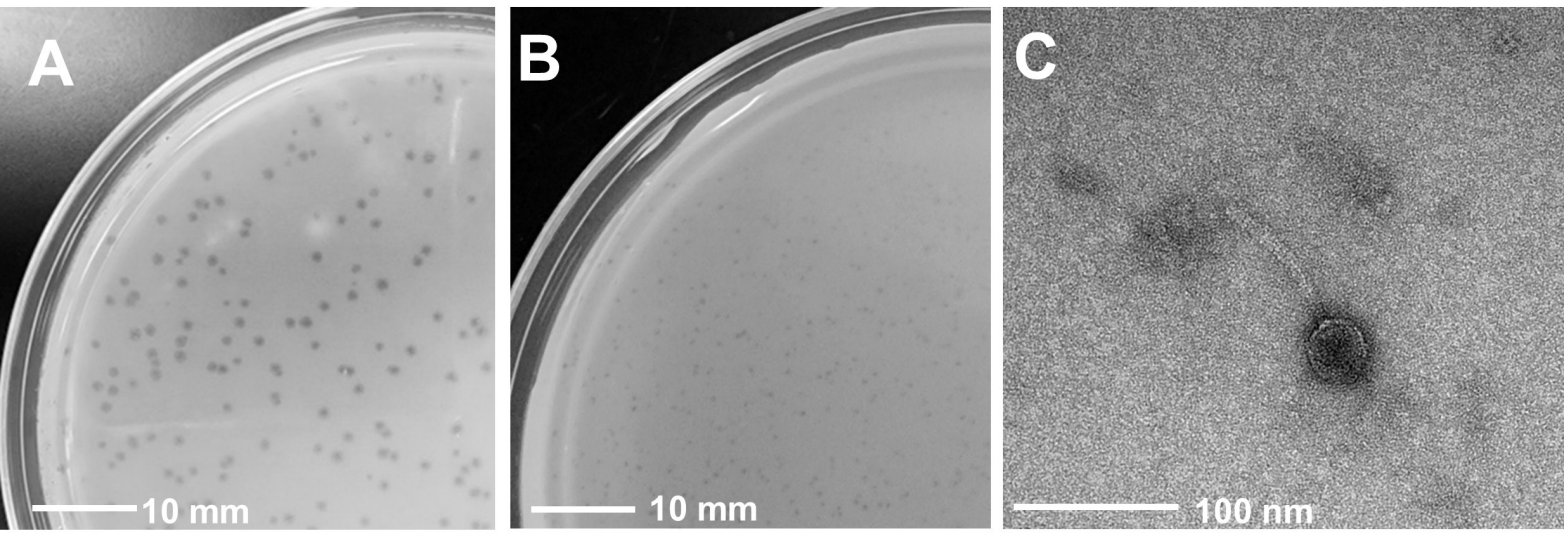
Piku (A) had consistent 1 mm (n=3) plaques while Utopia (B) ranged from 0.5 to 1 mm (n=3) plaques. Transmission electron microscopy (TEM) shows Utopia with an icosahedral capsid and flexible tail suggestive of a siphovirus morphology. (C) Both phages contain a tape measure protein in their genome which suggests the presence of a tail. Scale bars for plaque images and TEM micrographs are 10 mm and 100 nm, respectively. Utopia TEM image generated at the University of Maryland, Baltimore County.

## Description

Interest in using bacteriophages for clinical and agricultural applications, such as treating antibiotic-resistant infections or ensuring food safety, underscores the need to understand phage genomes and host interactions (Strathdee et al., 2023, Ranveer et al., 2024). To support this effort, the SEA-PHAGES program offers an effective platform for student researchers to contribute meaningfully to the expanding body of knowledge in phage biology (Heller et al., 2024).

Here, two bacteriophages, Piku and Utopia, were isolated using bacterial host *Arthrobacter globiformis* B-2979 and B-2880, respectively. Soil samples collected in Schaumburg and Oakbrook, Illinois (GPS coordinates in Table 1) were incubated with shaking in PYCa supplemented with cycloheximide at 22°C for 5 hours in the absence of the host bacterium (Zorawik et al., 2024). The filtered supernatants were plated in PYCa soft agar with *A. globiformis* B-2979 and *A. globiformis* B-2880, respectively and incubated for 48 hours at 22°C. Isolated phage plaques were purified a total of three times. Phages Piku and Utopia generated 0.5 – 1.0 mm (n=3) diameter plaques with clear centers and define edges ( Figure 1A, B). Phage lysates were imaged by negative stain (1% uranyl acetate) transmission electron microscopy (TEM) revealing a siphovirus morphology for Utopia; micrographs for Piku were of insufficient resolution to determine its structure ( Figure 1C ).

DNA was extracted from high titer lysates using Promega Wizard DNA Cleanup kit, libraries were constructed using the NEB Next Ultra II FS kit, and samples were sequenced for Piku (Illumina MiSeq 1000) and Utopia (Illumina NextSeq 1000). Single-end 150-base raw reads for Piku were assembled using Newbler (Miller, et al., 2010) and genome termini were identified using Consed v29 (Gordon and Green, 2013). Single-end 100-base raw reads for Utopia were trimmed with cutadapt 4.7 and filtered with skewer 0.2.2 prior to assembly with Newbler V2.9, as previously described (Russell, 2018). Sequencing data and genome characteristics, including length, GC%, and number of genes, are reported in Table 1. Piku and Utopia were assigned to Cluster FE based on gene content similarity of at least 35% to phages in the database PhagesDB (Russell and Hatfull 2017; Pope et al. 2017).

Genome annotation was performed using DNA Master V5.23.6 (Jacobs-Sera et al., 2014) with gene predictions from Glimmer (Delcher et al., 2007), and further refined using Starterator (Pacey, 2016), GeneMark v2.5 (Besemer and Borodovsky, 2005), and Phamerator v596 (Cresawn et al., 2011). Protein sequences were analyzed for similarity, transmembrane domains, and functions using the Actinobacteriophage and NCBI non-redundant database (Altschul et al., 1990), TMHMM v2 (Hallgren et al., 2011), and HHpred v2.08 with selected databases PDB_mmCIF70, SCOPe70, UnitProt-SwissProt-viral70, SMART_v6.0 (Soding et al., 2005). The presence of tRNAs were examined using the programs Aragorn and tRNAscanSE (Lowe and Eddy, 1997, Laslett and Canback, 2004). All software programs were used with their default setting.

Using the Gene Content Similarity (GCS) tool in PhagesDB, phage Piku and Utopia share 90.9% of their phams (clusters of phage proteins with high amino acid similarity) (Cresawn et al. 2011, Russell and Hatfull, 2017). Piku and Utopia encode 22 predicted genes, of which 15 and 14 genes, respectively, were assigned a putative function. No tRNAs were identified, consistent with the other 12 phages assigned to Cluster FE (Kotturi et al., 2024). All genes in both genomes are transcribed unidirectionally.

The left arm of the genome contains structural genes, followed by a putative lysis cassette consisting of an endolysin and two genes with predicted transmembrane domains (two and one domains, respectively) that may encode a holin. Based on the absence of identifiable lysogeny-related genes, both phages are predicted to follow a lytic replication cycle. The remaining genes encode diverse functions, including helix-turn-helix DNA-binding domain proteins, DNA methyltransferases, HNH endonucleases, and seven to eight proteins of unknown function. Notably, Piku is the only phage in the cluster known to encode a putative DNA methyltransferase.

**Nucleotide sequence accession numbers**

Piku is available through NCBI GenBank Accession number PQ362670 and sequence read archive (SRA) Number SRR33718586.

Utopia is available through NCBI GenBank Accession number PV876972 and sequence read archive (SRA) Number SRR33718576.

**Table 1: Soil collection site, average shotgun coverage, cluster assignment, and genome summary (size, ends, GC %, and number of genes).**

**Table:** 

**Phage name**	**Piku**	**Utopia**
**Location site (GPS)**	42.03 N, 88.10 W	41.83 N, 87.98 W
**Average shotgun coverage**	6511X	25899X
**Genome size (bp)**	15675 bp	15346 bp
**Genome termini**	3' single-stranded overhang 5’-CCACGGTCCCCGTCC-3’	3’ single-stranded overhang 5’-CCACGGTCCCCGTCC-3’
**GC content %**	64.2%	64.7%
**Number of Genes**	22	22
**Cluster**	FE	FE
